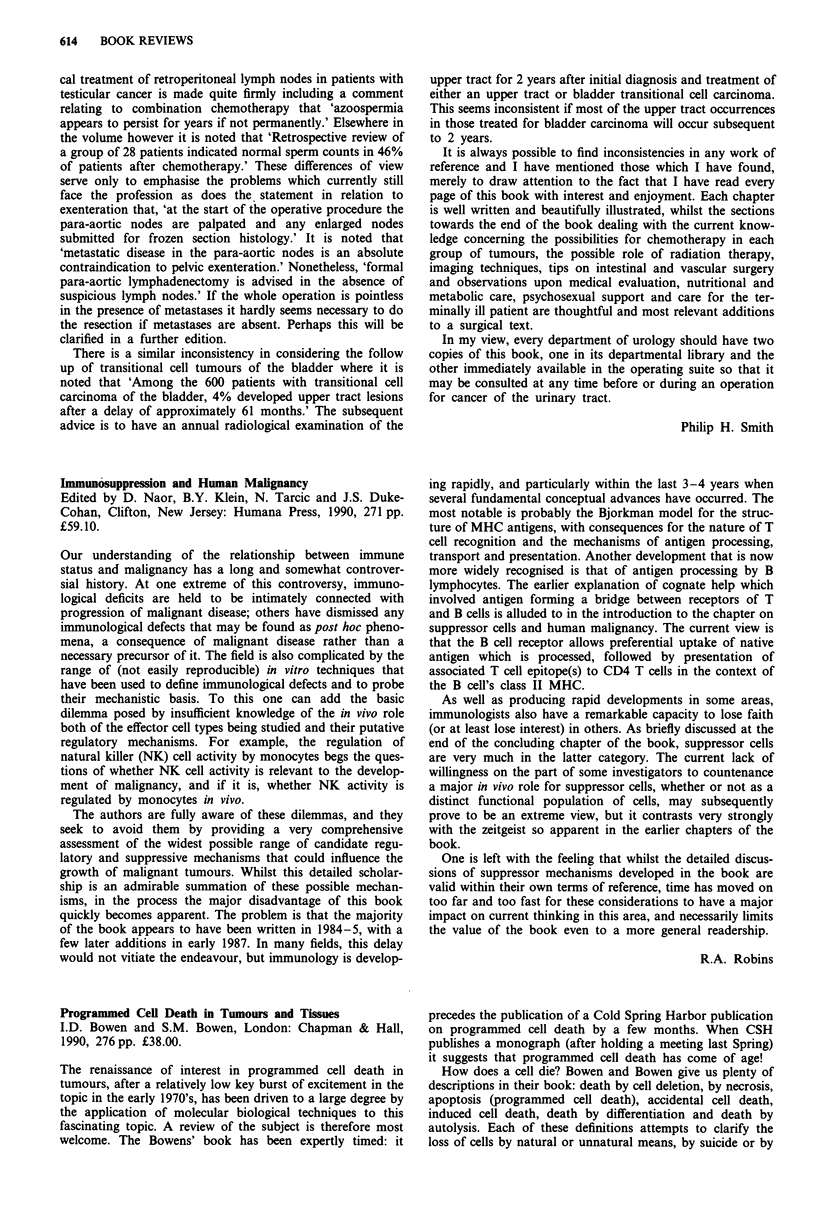# Immunosuppression and Human Malignancy

**Published:** 1991-09

**Authors:** R.A. Robins


**Immunosuppression and Human Malignancy**

Edited by D. Naor, B.Y. Klein, N. Tarcic and J.S. Duke-Cohan, Clifton, New Jersey: Humana Press, 1990, 271 pp. £59.10.

Our understanding of the relationship between immune status and malignancy has a long and somewhat controversial history. At one extreme of this controversy, immunological deficits are held to be intimately connected with progression of malignant disease; others have dismissed any immunological defects that may be found as *post hoc* phenomena, a consequence of malignant disease rather than a necessary precursor of it. The field is also complicated by the range of (not easily reproducible) *in vitro* techniques that have been used to define immunological defects and to probe their mechanistic basis. To this one can add the basic dilemma posed by insufficient knowledge of the *in vivo* role both of the effector cell types being studied and their putative regulatory mechanisms. For example, the regulation of natural killer (NK) cell activity by monocytes begs the questions of whether NK cell activity is relevant to the development of malignancy, and if it is, whether NK activity is regulated by monocytes *in vivo*.

The authors are fully aware of these dilemmas, and they seek to avoid them by providing a very comprehensive assessment of the widest possible range of candidate regulatory and suppressive mechanisms that could influence the growth of malignant tumours. Whilst this detailed scholarship is an admirable summation of these possible mechanisms, in the process the major disadvantage of this book quickly becomes apparent. The problem is that the majority of the book appears to have been written in 1984–5, with a few later additions in early 1987. In many fields, this delay would not vitiate the endeavour, but immunology is developing rapidly, and particularly within the last 3–4 years when several fundamental conceptual advances have occurred. The most notable is probably the Bjorkman model for the structure of MHC antigens, with consequences for the nature of T cell recognition and the mechanisms of antigen processing, transport and presentation. Another development that is now more widely recognised is that of antigen processing by B lymphocytes. The earlier explanation of cognate help which involved antigen forming a bridge between receptors of T and B cells is alluded to in the introduction to the chapter on suppressor cells and human malignancy. The current view is that the B cell receptor allows preferential uptake of native antigen which is processed, followed by presentation of associated T cell epitope(s) to CD4 T cells in the context of the B cell's class II MHC.

As well as producing rapid developments in some areas, immunologists also have a remarkable capacity to lose faith (or at least lose interest) in others. As briefly discussed at the end of the concluding chapter of the book, suppressor cells are very much in the latter category. The current lack of willingness on the part of some investigators to countenance a major *in vivo* role for suppressor cells, whether or not as a distinct functional population of cells, may subsequently prove to be an extreme view, but it contrasts very strongly with the zeitgeist so apparent in the earlier chapters of the book.

One is left with the feeling that whilst the detailed discussions of suppressor mechanisms developed in the book are valid within their own terms of reference, time has moved on too far and too fast for these considerations to have a major impact on current thinking in this area, and necessarily limits the value of the book even to a more general readership.

R.A. Robins